# Targeted therapy of irritable bowel syndrome with anti-inflammatory cytokines

**DOI:** 10.1007/s12328-021-01555-8

**Published:** 2021-12-04

**Authors:** Sunil Kumar, Priyanka Singh, Awanish Kumar

**Affiliations:** 1 Faculty of Bio-Sciences, Institute of Bio-Sciences and Technology, Shri Ramswaroop Memorial University, Lucknow- Deva Road, Barabanki, 225003 Uttar Pradesh India; 2 Department of Biotechnology, National Institute of Technology, Raipur, Chhattisgarh India

**Keywords:** Irritable bowel syndrome, Gut, Inflammation, Anti/pro-inflammatory cytokines, Therapy

## Abstract

Irritable bowel syndrome (IBS) is a multifactorial disease of which infection, as well as inflammation, has recently been considered as an important cause. Inflammation works as a potential pathway for the pathogenesis of IBS. In this review, we have discussed the targeted therapy of IBS. We used the search term “inflammation in IBS” and “proinflammatory” and “antiinflammatory cytokines and IBS” using PubMed, MEDLINE, and Google Scholar. The literature search included only articles written in the English language. We have also reviewed currently available anti-inflammatory treatment and future perspectives. Cytokine imbalance in the systematic circulation and the intestinal mucosa may also characterize IBS presentation. Imbalances of pro-and anti-inflammatory cytokines and polymorphisms in cytokine genes have been reported in IBS. The story of targeted therapy of IBS with anti-inflammatory cytokines is far from complete and it seems that it has only just begun. This review describes the key issues related to pro-inflammatory cytokines associated with IBS, molecular regulation of immune response in IBS, inhibitors of pro-inflammatory cytokines in IBS, and clinical perspectives of pro- and anti-inflammatory cytokines in IBS.

## Introduction

Irritable bowel syndrome (IBS) is a functional gastrointestinal (GI) disorder characterized by abdominal pain or discomfort, flatulence, and irregular bowel habits. The worldwide prevalence of IBS is about 11% with more prominent in women [1]. The inflammatory role of cytokines cannot be ignored in patients with IBS. As the fact arises that an imbalance between pro-and anti-inflammatory cytokines plays an important role in the pathogenesis of IBS, which has been substantiated by several studies worldwide [2]. Pro- and anti-inflammatory cytokines are important modulators of the immune response and they also play a role in intestinal inflammation [3]. Cytokine production is under genetic control and an imbalance in the level of cytokine secretion may cause disease susceptibility along with clinical symptoms [4, 5]. In different studies, the balance of pro-inflammatory and anti-inflammatory responses and the mediators that are involved in the complex interactions have been reported [6, 7]. There is evidence of sustained inflammation in IBS supported by the number of studies that have detected a low level of anti-inflammatory cytokines in patients with IBS [8] or others that found high levels of those pro-inflammatory or a misbalance of the pro-and anti-inflammatory cytokine proportion [9, 10]. The complex dialogue between the structures involved in maintaining the homeostasis includes interrelation of nervous, immune, and endocrine systems [11] where a pivotal piece is a brain that governs the humoral and neurological systems [12, 13] in a complex network with multidirectional communicating systems. A secretion of tumour necrosis factor-*α* (TNF-*α*) as a pro-inflammatory cytokine is associated with a single-nucleotide polymorphism (SNP) in the promoter region of the TNF-*α* gene [14, 15]. The normal gastrointestinal immune response is under tight regulation with the balance between pro- and anti-inflammatory cytokines or mediators defining the immune status of the gut [16].

It has been studied that IBS is a multifactorial disease. IBS involves genetic as well as environmental factors, especially psychological distress that seems to predispose to IBS [17, 18]. The process of alteration in systemic or mucosal cytokines, as well as cytokine gene polymorphisms, seems to have a significant role in the development of IBS [11]. The pathophysiology of this disorder has been attributed to several conditions such as gut inflammation, altered visceral sensitivity, food sensitivities, gene susceptibility, and changes in the gut–host microbiome, and also altered brain–gut signaling. The current review targets the treatment options of IBS based on anti-inflammatory cytokines.

**Fig. 1:**
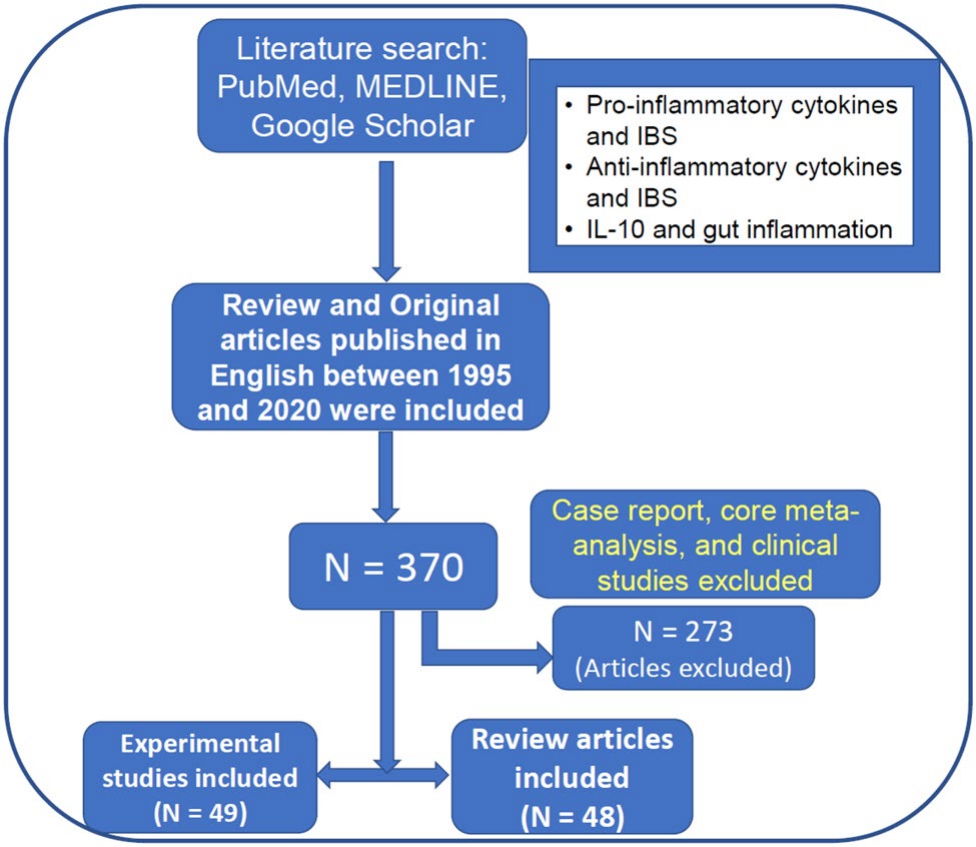
Flowchart of inclusion and exclusion of published articles for writing the present manuscript

We searched the literature using PubMed, MEDLINE, and Google Scholar with related key terms [pro-inflammatory cytokines and IBS, anti-inflammatory cytokines Interleukin-10 (IL-10) and IBS, IL-10 and gut inflammation], and prepared this review article on that basis. The reference lists of all identified articles were searched manually for additional information. Review and original papers published in English between 1995 and 2020 were included in this article. Articles that were case reports, core meta-analysis, and clinical studies were excluded. A total of 370 articles were identified in the initial search; 273 articles were excluded, because they did not meet the criteria for inclusion in this review, and 97 articles (49 experimental studies and 48 reviews) were included in this review (Fig. 1).

## Pro-inflammatory cytokines associated with IBS

Cytokines are the immunological component that plays an important role in signaling and are secreted by the immune cell and they affect the other cells as they link T helper cells (TH1 & TH2) and cytotoxic T lymphocytes (Tc cells) [19]. Cytokines also play an important modulator in intestinal inflammation [3]. Some of these cytokines decrease the risk of IBS such as anti-inflammatory cytokines as IL-10 [20, 21], and some of them are found associated with the development of the IBS [22] such as pro-inflammatory cytokines, which always promote the inflammation response such as IL-1, IL-6, IL-8, IL-12, IL-18, and TNF-α, etc. [23–25]. The main pro-inflammatory cytokines associated with IBS are IL-6, IL-8, and TNF-α [26]. In some studies, it is found increased levels of IL-6, IL-8, IL-12 (in Mexican children), and TNF-α as well as decreased levels of IL-10 (an anti-inflammatory cytokine) in patients with IBS than healthy controls [22, 27, 28].

IL-6 is multifunctional cytokine, secreted by macrophages, adipocytes, and other immune cells like fibroblasts as well as endothelial cells and regulates the immune response as like hematopoiesis and acute-phase reactions, and may play a key role in defense mechanisms of the host [29]. While the TNF-α multifunctional pro-inflammatory cytokines are mainly secreted by the macrophage and in some amount by monocytes and play an important role in chronic inflammation. It facilitates the alteration of properties of vascular epithelial cells and activation of neutrophils. Due to the regulation of other cytokines, it is also known as the “Master-regulator” cytokine [30]. IL-8 which is also known as the “Chemokines CXCL8” [31] secreted by macrophages and other cells such as endothelial cells, epithelial cells, and airway smooth muscles cells [32] was higher in patients with IBS. It begins and intensifies the chronic inflammation and acute-phase inflammatory response. Increased level of IL-8 does not influence the level of IL-10 [33]. Hence, it could be a potential marker of inflammation in IBS.

## Molecular regulation of immune response in IBS

In several clinical studies, it has been found that intestinal permeability is increased throughout the intestine [34, 35]. These studies aimed at unraveling the molecular basis of this phenomenon in IBS are mainly focused on the large bowel [36, 37]. The application of high-throughput technologies including microarray gene profiling of mucosal samples taken from IBS patients has provided meaningful insights into the complex network of molecular interactions regulating the intestinal barrier function as well as its relation to clinical manifestations of IBS [38]. It has been studied that the colonic transcriptomic signatures of IBS patients have significant changes in the expression of a wide variety of genes that are involved in the impairment of mucosal immune response to microbial pathogens [39]. Barrier dysfunction in IBS has been linked to the down-regulation of the tight junctions (TJ) scaffolding protein zonal occludins (ZO-1) [40] and also proteasome-mediated occludin degradation in the colonic mucosa of IBS patients [41]. In protein down-regulation, the structural and functional modulation of barrier function can also be achieved by endocytosis of TJ proteins by a mechanism that depends on the stimulus involved [42]. Pro-inflammatory factors such as TNF and bacterial products were reported for having properties to induce the internalization of ZO-1 and occludin by caveolar-mediated endocytosis [43] together with a concomitant loss of barrier function. It has been recently reported that diarrhea-predominant IBS (IBS-D) patients harbour a distinctive miRNA profiling which reflects an elevated transcription regulation activity that may account for some of the pathological acts observed in this disorder such as distorted barrier function, low-grade inflammation, as well as immune activation [36, 44]. Previously also reported that small bowel and colon tissues of IBS-D patients with increased intestinal permeability may show a consistent molecular profile that is involved in up-regulation of miR-29a (miRNA) and down-regulation of glutamine synthetase expression when compared to healthy and IBS-D with normal permeability [45]. The molecular alteration in the immune response of IBS is shown in Table 1. Previously, it has been reported that IL-10 down-regulates the cytokines IL-1 beta and TNF-alpha secretion and also messenger RNA levels in patients’ peripheral monocytes. Also, IL-1 receptor antagonist secretion is induced, and hence, IL-10 can restore diminished in vitro IL-1 receptor antagonist/IL-1 beta ratios in patients to normal levels. Furthermore, equal concentrations of IL-10 were detectable in intestinal lamina propria biopsy homogenates of both normal and patient with IBS. In vitro release of pro-inflammatory cytokines from patient's peripheral monocytes is dramatically down-regulated [46].

**Table 1:** Molecular alteration in IBS

Alterations	Findings in IBS patients	Barrier dysfunction mechanism	References
Molecular level	In the colonic mucosa, proteasome-mediated occludin degradation occur	Increased proteasome activity induced by pro-inflammatory cytokines resulting in loss of TJ integrity	[41]
	In both small and large bowel of IBS-D, the process of up-regulation of miR-29a takes place	Down-regulation of glutamine synthetase expression	[45]
	In both small as well as large bowel mucosa of IBS-D there is reduced claudin 1 and claudin 4 protein levels	Barrier function weaken	[72]
	In both the jejunal and colonic mucosa down-regulation and redistribution of ZO-1 occur	Impaired claudin recruitment, TJ formation, and development of barrier function	[40, 73]

**Fig. 2:**
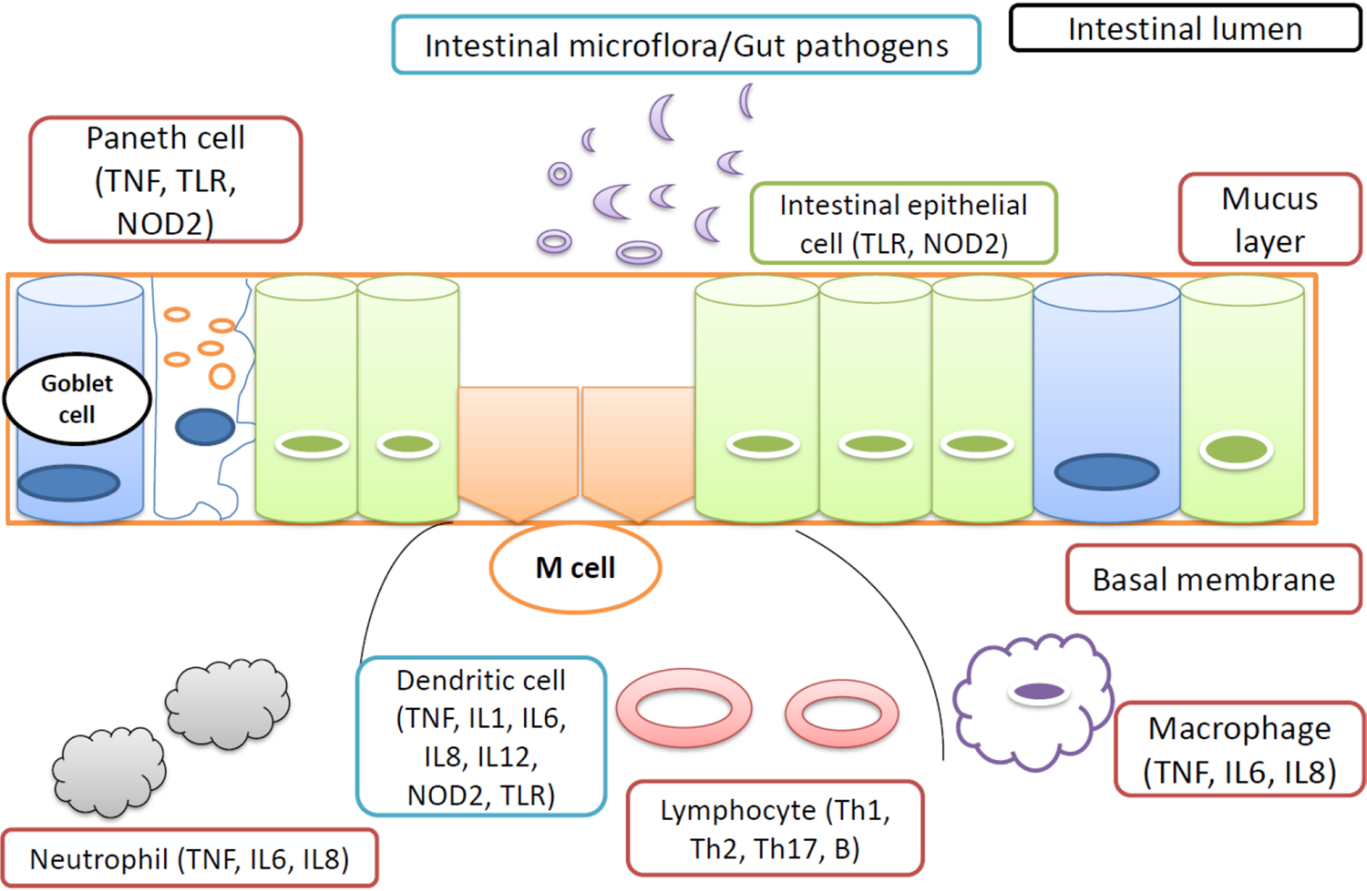
Molecular mechanism of inflammation in IBS

The intestinal barrier as shown in Fig. 2 consists of a bacterial biofilm that is a layer of mucus and intestinal epithelium in which lies the innate immune system involving dendritic cells, paneth cells, macrophages, and neutrophils [47, 48]. The intestinal epithelial barrier works to protect the body from several potential bacterial threats. However, the number of goblet cells secreting mucins constitutes the protective mucus in the intestinal epithelium [49]. This process of recognition of pathogenic bacterial components is performed via Toll-like receptor (TLR) and nucleotide oligomerization domain (NOD) receptors [50]. Thus, the inflammatory reaction involves mucosal immune system activation concerning mononuclear cells, lymphocytes, as well as dendritic cells of the subepithelial dome. Hence, we can say that inflammation is mediated by cytokines (TNF, IL) secreted by these different immune cells.

Inflammation may have a role in IBS pathogenesis and the development of IBS symptoms in a subgroup of individuals, according to new research. Colonoscopic biopsy specimens from individuals who met the Rome criteria for clinical diagnosis of IBS have been studied earlier [51]. The number of activated immunocompetent cells in the intestinal mucosa increased, including T lymphocytes, neutrophils, and mast cells, suggesting a role for the mucosal immune system in pathogenesis. Following investigations found an increased frequency of numerous surrogate indicators for inflammation in patients with IBS, with the most consistent finding being an increased number of mast cells in IBS patients' GI tracts [40, 52–54]. Mast cells are linked to wound healing, pathogen defense, and hypersensitivity in the gastrointestinal mucosa. They degranulate to produce inflammatory and immunological mediators, which attract additional inflammatory cells to the gastrointestinal mucosa. Increased mast cells in IBS patients have been linked to specific symptoms of IBS, such as bloating and stomach discomfort, according to many studies [54, 55]. The presence of activated T lymphocytes in mucosal biopsy specimens from IBS patients is another finding [11, 52, 54]. Several investigations have found that lymphocyte infiltration in the myenteric plexus of patients is higher than in healthy controls [40, 54, 56]. In addition, IBS patients' colonic biopsies and blood samples include more activated T cells [57]. T lymphocytes have a role in adaptive immunity and perform a variety of tasks, including activating B lymphocytes and macrophages and destroying contaminated host cells [36]. In addition, increased pro-inflammatory cytokine expression in peripheral blood mononuclear cells [58] and serum [59] may indicate a proclivity for immunological activation in IBS patients. The findings supporting the function of inflammatory and proinflammatory cytokines in IBS have been reviewed in the subsequent headings.

## Inhibitors of pro-inflammatory cytokines in IBS

Pro-inflammatory cytokines are produced predominantly by activated macrophages and are involved in the up-regulation of inflammatory reactions. IL-1β, IL-6, and TNF-α are the typical pro-inflammatory cytokines [60, 61]. However, there are several other cytokines which cause inflammations in IBS are given in Table 2. The cytokine inhibitors are used to explain a heterogeneous group of drugs, which are involved in the activity such as decrease in the synthesis of cytokines, decrease in its concentration in the free active form block its interaction with specific receptors, and also interfere with the signaling of cytokine receptors [62]. The cytokine inhibitors are general cytostatic drugs such as azathioprine or methotrexate supposed to be immune-suppressants or anti-inflammatory agents. A cytostatic drug with a higher selectivity for immune cells is mycophenolate [63]. In further studies, it has been reported that muromonab CD3 decreases selectively the circulating pool of this lymphocyte subpopulation predominantly by complement lysis and acts as an immunosuppressive that is a cytokine inhibitor [64]. Furthermore, the glucocorticoids prednisone has anti-inflammatory or immunosuppressive effects also act as cytokine inhibitors [65]. Glucocorticoids have the property to bind to the glucocorticoid receptor in the cytoplasm of cells by releasing it from binding to the heat shock protein (HSP), whereas Infliximab, which is a humanized monoclonal antibody, has been used against TNF [66].

**Table 2:** List of cytokines and their activities

S.N	Cytokines	Major activities	References
1	IL-4	Inhibition of LPS-induced pro-inflammatory cytokines synthesis as well as promotes Th2 lymphocyte development	[74]
2	IL-6	Inhibition of TNF as well as IL-1 production by macrophages	[75]
3	IL-10	Inhibition of TH1-type lymphocyte responses and inhibition of monocyte or macrophage and neutrophil cytokine production	[51]
4	IL-11	Inhibits pro-inflammatory cytokines response by monocyte or macrophages and also promotes Th2 lymphocyte response	[51]
5	IL-13	Shares homology with IL-4 and also shares IL-4 receptor; attenuation of monocyte or macrophage function	[76]
6	II-1ra	Specific inhibitor of IL-1α- and IL-1β-mediated cellular activation at the IL-1 cellular receptor level	[77]
7	TGF-α	A major human serum factor that promotes human keratinocyte migration	[78]
8	TGF-β	Inhibition of monocyte or macrophage MHC, class II expression, and pro-inflammatory cytokines synthesis	[51]

## Clinical perspectives of pro- and anti-inflammatory cytokines in IBS

**Fig. 3:**
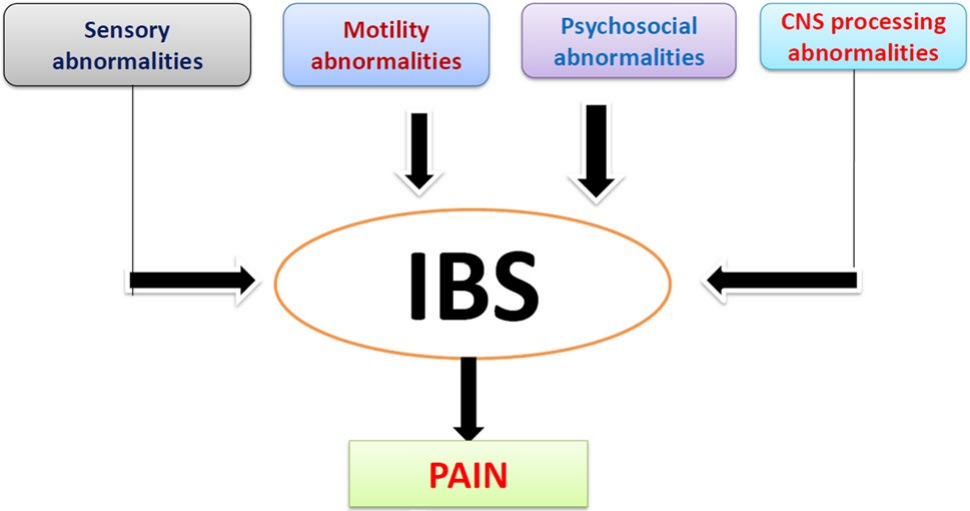
Bio-psychosocial model of IBS

The epidemiological and clinical perspectives of IBS may vary with geographical regions due to several factors such as differences in diet, gastrointestinal infection, and infestations; it also includes socio-cultural and psychosocial factors, religious, and illness beliefs as well as symptom perception and reporting [67] (Fig. 3). Previously, it has been studied that the drugs available for the treatment of IBS have only a modest effect on symptom improvement and may not alter the natural history of the condition [68, 69]. In the context of clinical perspectives, the management strategy recommends a positive diagnosis, development of a good doctor–patient relationship as well as identification of contributing factors, critical appraisal of the efficacies of various drugs according to the subtype of IBS, and continuing care [67, 70]. There is no test to definitively diagnose IBS. The currently used drugs for the treatment of IBS are shown in Table 3.

**Table 3:** Drug or medication used for the treatment of IBS with their mechanism of action

S.No	Drug/medication	Mechanism of action	References
1	Amitriptyline, desipramine, trimipramine, imipramine, doxepin	Serotonin reuptake inhibitors that increase central serotonin and norepinephrine synaptic concentrations which results in decreased motility and visceral pain	[79]
2	Citalopram, paroxetine, fluoxetine	Selective serotonin reuptake inhibitors that increase central serotonin synaptic concentrations, which decreases motility in patients with IBS-D and increases motility in IBS-C; serotonin reuptake inhibition, decreases visceral pain	[80]
3	Clonidine	Central α-agonist that reduces sympathetic outflow and results in decreased motility	[81]
4	Dicyclomine	Non-specific antimuscarinic and direct antispasmodic that results in relaxation of intestinal smooth muscle	[82]
5	Hyoscyamine	Non-specific antimuscarinic that results in relaxation of intestinal smooth muscles; decreases gastric acid secretions	[83]
6	Ketotifen	Selective, non-competitive mast cell stabilizer that results in decreased inflammatory response	[84]
7	Lubiprostone	Chloride channel-2 activator that increases chloride and intestinal fluid secretion, which increases motility and decreases transit time, mucosal membrane stabilization may also reduce inflammation and sensitization	[85]
8	Linaclotide	Guanylate cyclase-C agonist that increases intra- and extracellular cyclic guanosine monophosphate (cGMP) concentrations, resulting in increased chloride, bicarbonate, and fluid secretion in the intestinal lumen; the increased intestinal fluid decreases transit time and the increased extracellular cGMP decreases visceral pain	[86]
9	Loperamide	Opioid receptor agonist that decreases gut motility, fluid secretion, increases anal sphincter tone, resulting in increased transit time, decreased fecal volume	[87]
10	Octreotide	Somatostatin analogue that decreases visceral sensitivity, gastric acid and fluid secretion; mechanism not fully understood	[88]
11	Pregabalin	GABA analogue that binds directly to α2δ centrally which results in decreased visceral pain; mechanism not fully understood	[89]
12	Peppermint oil	Menthol impairs calcium transmembrane transit and thereby relaxes intestinal smooth muscle	[90]
13	Psyllium, bran	Fiber absorbs water into the intestine, creating a viscous fluid that increases motility and decreases transit time	[91]
14	Rifaximin	Non-absorbable, broad-spectrum, gut-selective antibiotic that stabilizes gut flora and prevents overgrowth	[92]

The roles of pro-inflammatory activities of TNF-α are well established in IBS. Several medications are currently in the research pipeline for the treatment of IBS [15, 71]. There are medications available in certain countries that are effective and used for the treatment of IBS including ramosetron [72]. A review of clinicaltrials.gov using the search terms ‘irritable bowel syndrome’ and ‘IBS’ has identified 22 investigational drugs for the treatment of IBS with the ongoing investigation into their utility as a potential treatment [73]. Some of the results of the clinical trial are shown in Table 4. In further studies, variations in the circulating pro-inflammatory IL-6 levels and IL-6 gene polymorphisms have been demonstrated in IBS [9]. In several studies, the cytokine profiles of IBS-D patients and healthy individuals have been reported. Scully et al. (2010) have reported that the presence of elevated serum IL-6 and TNF- α in IBS-D patients as compared to controls signifies ongoing mild inflammation [74]. As a result, it was found that pro-inflammatory cytokines in female IBS patients with extraintestinal co-morbidities showed increased levels of IL-6, IL-8, and TNF-α [75]. Similarly, Kennedy et al. (2014) also reported elevated cytokine levels in patients suffering from IBS indicating some immune dysregulation in these patients [76]. Pro-inflammatory cytokines IL-6 and TNF-α have been shown to control the release of corticotrophin-releasing hormone, the main hypothalamic regulatory peptide of the hypothalamic–pituitary–adrenal (HPA) axis [59, 77]. Thus, an increase in these cytokines as seen in IBS patients may be involved in exaggerated activation of the HPA axis. This may be due to enhanced cellular immune response with increased pro-inflammatory cytokine production in IBS-D [58]. It has been previously seen that IL-6 and TNF-α cytokine gene polymorphism could change an individual’s susceptibility to IBS and they have a pathophysiological role [75]. This study demonstrated that patients with IBS-D can have increased levels of pro-inflammatory cytokines IL-6 and TNF-α with no difference in anti-inflammatory cytokine levels of IL-10 [21]. In previous studies of Scully et al. (2010), they reported that increased plasma levels of IL-6 and IL-8 were found in patients with IBS [74], while a study by Dinan et al. (2006) reported similar findings along with no variations in the anti-inflammatory cytokine IL-10 levels [59]. Furthermore a study by Schmulson et al. (2012) observed that at least some patients suffering from IBS produce lower amounts of the anti-inflammatory cytokine IL-10 [8]. A study from India by Rana et al. (2012) reported the IL-6 and TNF-α levels were found to be higher in IBS-D with no change in the IL-10 level [26]. In concordance with the above, Bashashati et al. (2017) also reported the same findings [9]. Hence, it is well established that pro-inflammatory cytokines such as IL-6 and IL-8 were found to be higher in patients with IBS, whereas anti-inflammatory cytokine IL-10 was found to be low[21]. However, it is quite evident that there is no difference in the levels of IL-6 between controls and IBS, which is in contrast to many previous studies.

**Table 4:** Clinical trials with treatment

S.N	Disease	Condition characterized	Intervention/treatment	Study Phase	Reference
1	IBS	By constipation	Drug: Placebo	3	[93]
2		By constipation	Drug: Plecanatide	3	[94]
3		By diarrhoea	Drug: Rifaximin	3	[95]
4		By stress	Behavioral: Stress Management and Resilience Training Program (SMART)	Not applicable	[96]
5		By stress	Behavioral: Self-Management Stress Reduction Program (SMSR)	Not applicable	[97]

In comparison to IBS patients or controls, the intensity of TNF-α was shown to be lower in Inflammatory Bowel Disease (IBD) patients. Several additional investigations have found higher TNF-α levels in the mucosa of IBD patients [78], whereas others have been unable to identify increased TNF-α levels [79, 80]. IL-1β levels in the mucosa were also lower in IBD patients compared to IBS patients or controls. IL-6 mucosal levels, on the other hand, were higher in IBD patients than in the other groups, which are consistent with many studies that have identified IL-6 as a significant component in IBD [81, 82].

Furthermore, the fact that TNF-α mucosal levels were higher in IBS patients compared to IBD patients or controls is intriguing, indicating that TNF-α may play a role in IBS aetiology. Given the verified rise in IBD patients, as shown in numerous publications [3], more study into TNF-α serum levels in IBS patients is likely to yield more information. The levels of IL-6 and IL-1β were similar to those found in controls, indicating that they may have a minor role in IBS. TNF-α levels were found to be somewhat different in IBS subgroups based on symptomatology, with a decrease in IBS-D patients and an increase in IBS-C patients, but no changes in IL-6 or IL-1β.

TNF-α, IL-6, and IL-1β levels were higher in unprotected controls, which might be attributed to normal intestinal inflammation [83]. Natural intestinal inflammation is a normal reaction that protects the gut from damage by precisely fitting into a variety of pro-inflammatory stressors, and is thus necessary for homeostasis. The gut grows and works in the presence of a complex and massive microbial load as well as a constant supply of food antigens, resulting in a state of “normal inflammation”. In normal controls, anti-inflammatory cytokines like IL-10 may help to reduce inflammatory processes.

## Post-infectious IBS (PI-IBS): a cause of IBS

Post-infectious IBS (PI-IBS) research has offered etiological insights into IBS aetiology. It is widely known that after infective gastroenteritis, more than 10% of those who are afflicted develop PI-IBS [84]. When bacterial gastroenteritis [85] is compared to viral gastroenteritis, the risk of PI-IBS appears to be higher [86]. In rectal samples collected 3 months after infection in individuals with PI-IBS, increased expression of IL-1β mRNA has been observed [87]. Patients who did not develop PI-IBS did not show this. The pro-inflammatory cytokine IL-1β is responsible for cellular inflammation. In patients with PI-IBS, serial rectal biopsies indicated chronically elevated T cells and other enteroendocrine cells, compared to those whose symptoms ultimately abate [88]. The CD3 + , CD4 + , and CD8 + T-cell counts in the gastrointestinal mucosa of patients who had PI-IBS were also considerably greater [88]. The gut's adaptive immune response is mediated by these cells. These data, taken together, point to an inflammatory–immune etiopathogenesis of IBS.

The disturbance of normal gut flora is another effect of infective gastroenteritis; however, the gut microbiota during parasite infection has yet to be investigated [84]. Studies using 16S-rRNA sequencing have shown lower microbial diversity in patients with PI-IBS. Although the gut microbiota is known to vary from person to person, an increase in *Bacteroides* and *Prevotella* bacteria has been found in individuals with IBS when compared to healthy controls [89]. Inflammation is considered to be modulated by the microbiome, which can act directly or indirectly through microbial metabolites. Microbial dysbiosis has been found to increase inflammation [90] and impede normal lymphocyte function [91], sustaining low-grade chronic inflammation.

## Conclusions

Inflammation plays an important role as a pathway involved in the pathogenesis of IBS. To date in the field of functional gastrointestinal disorders, a high number of studies have been done. Several studies focused on the role of cytokines as well as cytokine-producing gene polymorphisms in IBS are very limited. Many more studies having large sample sizes including the molecular ones are used to evaluate the role of various cytokines apart from the established ones in IBS patients. This will be involved in the development of a better understanding of the etiopathogenesis, targeted therapy, and better management of IBS. Stress and psychological symptoms can alter cytokine profiles and pro-inflammatory cytokines which could be higher in those suffering from co-morbid anxiety and depression. Cytokine patterns in IBS with co-morbid fibromyalgia, premenstrual syndrome, and chronic fatigue syndrome are different as compared to patients suffering from IBS only. A trend appears to exist as in the presence of high levels of pro-inflammatory cytokines such as TNF- α, IL-1β, IL-6, IL-8, and diminished levels of the anti-inflammatory cytokine that is IL-10. IL-10 is a regulatory cytokine that inhibits the release of pro-inflammatory cytokines as well as antigen presentation. Therefore, we advocate IL-10 as a potent anti-inflammatory biological therapy as well as cytokine of interest for IBS and it could be interesting to further explore it more for IBS especially in IBS-D. The persistence level of low-grade intestinal inflammation in patients suffering from IBS may be the result of a higher ratio or percentage of pro-inflammatory cytokines or lower ratio or percentage of anti-inflammatory cytokines that give rise to an imbalance in the activity of cytokines. Hence, we can conclude that targeting an increase in IL-10 level could lead to a possible treatment option for a subset of patients with IBS particularly IBS-D.
